# 2,5-Dichloro­anilinium chloride monohydrate

**DOI:** 10.1107/S1600536809004395

**Published:** 2009-02-13

**Authors:** B. Thimme Gowda, Sabine Foro, B. S. Saraswathi, Hiromitsu Terao, Hartmut Fuess

**Affiliations:** a Department of Chemistry, Mangalore University, Mangalagangotri 574 199, Mangalore, India; b Institute of Materials Science, Darmstadt University of Technology, Petersenstrasse 23, D-64287 Darmstadt, Germany; c Faculty of Integrated Arts and Sciences, Tokushima University, Minamijosanjima-cho, Tokushima 770-8502, Japan

## Abstract

The title compound, C_6_H_6_Cl_2_N^+^·Cl^−^·H_2_O, is composed of discrete cations, choride anions and water mol­ecules, which are connected through N—H⋯Cl, O—H⋯Cl and N—H⋯O hydrogen bonding. Two H atoms of the positively charged –NH_3_^+^ group have two chloride acceptors and the other one has the O atom of the water mol­ecule as acceptor. The chloride anions form hydrogen bonds with two H atoms from two different water mol­ecules and two H atoms from two positively charged –NH_3_^+^ groups.

## Related literature

For water-free 2,5-dichloro­anilinium chloride see: Gray & Jones (2002 ▶).
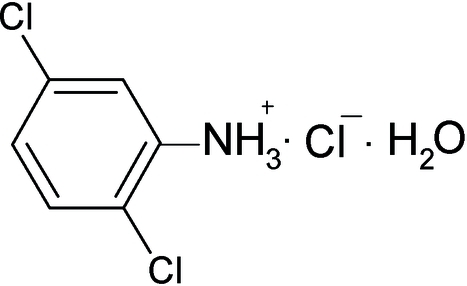


## Experimental

### 

#### Crystal data

C_6_H_6_Cl_2_N^+^·Cl^−^·H_2_O
*M* *_r_* = 216.48
Monoclinic, 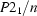

*a* = 7.679 (1) Å
*b* = 6.476 (1) Å
*c* = 19.060 (5) Å
β = 96.95 (3)°
*V* = 940.9 (3) Å^3^
*Z* = 4
Cu *K*α radiation
μ = 8.39 mm^−1^
*T* = 299 (2) K
0.35 × 0.30 × 0.10 mm

#### Data collection

Enraf–Nonius CAD-4 diffractometer
Absorption correction: ψ scan (North *et al.*, 1968 ▶) *T*_min_ = 0.109, *T*_max_ = 0.432
3331 measured reflections
1669 independent reflections
1421 reflections with *I* > 2σ(*I*)
*R*_int_ = 0.068
3 standard reflections frequency: 120 min intensity decay: 1%

#### Refinement

*R*[*F* ^2^ > 2σ(*F* ^2^)] = 0.049
*wR*(*F* ^2^) = 0.143
*S* = 1.10
1669 reflections
125 parameters
3 restraints
Only H-atom coordinates refined
Δρ_max_ = 0.39 e Å^−3^
Δρ_min_ = −0.49 e Å^−3^

### 

Data collection: *CAD-4-PC* (Enraf–Nonius, 1996 ▶); cell refinement: *CAD-4-PC*; data reduction: *REDU4* (Stoe & Cie, 1987 ▶); program(s) used to solve structure: *SHELXS97* (Sheldrick, 2008 ▶); program(s) used to refine structure: *SHELXL97* (Sheldrick, 2008 ▶); molecular graphics: *PLATON* (Spek, 2003 ▶); software used to prepare material for publication: *SHELXL97*.

## Supplementary Material

Crystal structure: contains datablocks I, global. DOI: 10.1107/S1600536809004395/bt2865sup1.cif

Structure factors: contains datablocks I. DOI: 10.1107/S1600536809004395/bt2865Isup2.hkl

Additional supplementary materials:  crystallographic information; 3D view; checkCIF report

## Figures and Tables

**Table 1:** Hydrogen-bond geometry (Å, °)

*D*—H⋯*A*	*D*—H	H⋯*A*	*D*⋯*A*	*D*—H⋯*A*
N1—H11⋯Cl3^i^	0.92 (4)	2.24 (4)	3.123 (3)	162 (3)
N1—H12⋯Cl3	0.94 (4)	2.16 (4)	3.099 (3)	172 (3)
N1—H13⋯O1^ii^	0.88 (4)	1.82 (4)	2.699 (4)	175 (4)
O1—H1*A*⋯Cl3	0.85 (3)	2.37 (3)	3.212 (3)	172 (4)
O1—H1*B*⋯Cl3^iii^	0.83 (3)	2.34 (3)	3.158 (3)	169 (4)

Symmetry codes: (i) 
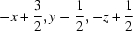
; (ii) 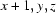; (iii) 
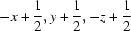
.

**Fig. 1.**
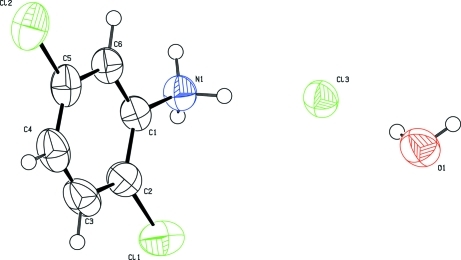
Molecular structure of the title compound, showing the atom labeling scheme. The displacement ellipsoids are drawn at the 50% probability level. The H atoms are represented as small spheres of arbitrary radii.

**Fig. 2.**
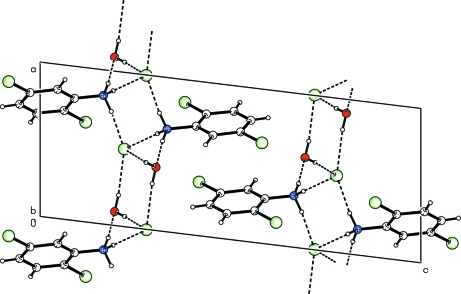
Molecular packing of the title compound with hydrogen bonding shown as dashed lines.

## supplementary crystallographic information

### Comment

The crystal structure of water free 2,5-dichloroanilinium chloride has been reported (Gray & Jones, 2002). We report herein the crystal structure of 2,5-dichloroanilinium chloride monohydrate. The title compound showed interesting H-bonding in its crystal structure (Fig. 1). Two H-atoms of the positively charged NH_3_ group have two chloride acceptors and the other H has O atom acceptor of the water molecule, while chloride anions are linked by four-center hydrogen bonds, with each chloride forming H-bonding with two H-atoms, one each from two different water molecules and two H-atoms, one each from two positively charged NH_3_ groups. This is in comparison with the usual set of hydrogen bonds from NH_3_ to chloride leading to layer structure observed with water free 2,5-dichloroanilinium chloride (Gray & Jones, 2002), with a short Cl1..Cl3 contact. Further, the water free structure involved four weak interactions, namely the three hydrogen bonds H4···Cl3, H6···Cl2 and H3···Cl1 and the chlorine-chlorine interaction Cl2···Cl3. The crystal packing of (I) through N—H···Cl, O—H···Cl and N—H···O hydrogen bonding (Table 1) is shown in Fig.2

### Experimental

The solution of pure 2,5-dichloroaniline (0.02 mole) in ethanol (20 cc) was treated dropwise with dilute hydrochloric acid (>0.025 mole) with constant stirring. The resulting mixture was slowly evaporated at room temperature to obtain 2,5-dichloroanilinium hydrochloride monohydrate. The resultant solid was recrystallized to constant melting point from ethanol. The single crystals used in X-ray diffraction studies were grown in ethanolic solution by slow evaporation at room temperature.

### Refinement

H atoms were located in a difference map, and their positional parameters were refined freely except for the water H atoms which were refined with the O—H distances restrained to 0.85 (4) Å and H—H distance restrained to 1.37 (4)Å. All H atoms were refined with isotropic displacement parameters set to 1.2 times of the *U*_eq_ of the parent atom.

### Crystal data

**Table:**

C_6_H_6_Cl_2_N^+^·Cl^−^·H_2_O	*F*(000) = 440
*M**_r_* = 216.48	*D*_x_ = 1.528 Mg m^−^^3^
Monoclinic, *P*2_1_/*n*	Cu *K*α radiation, λ = 1.54180 Å
Hall symbol: -P 2yn	Cell parameters from 25 reflections
*a* = 7.679 (1) Å	θ = 6.0–20.3°
*b* = 6.476 (1) Å	µ = 8.39 mm^−^^1^
*c* = 19.060 (5) Å	*T* = 299 K
β = 96.95 (3)°	Plate, colourless
*V* = 940.9 (3) Å^3^	0.35 × 0.30 × 0.10 mm
*Z* = 4	

### Data collection

**Table:**

Enraf–Nonius CAD-4 diffractometer	1421 reflections with *I* > 2σ(*I*)
Radiation source: fine-focus sealed tube	*R*_int_ = 0.068
graphite	θ_max_ = 67.2°, θ_min_ = 4.7°
ω/2θ scans	*h* = −9→9
Absorption correction: ψ scan (North *et al.*, 1968)	*k* = 0→7
*T*_min_ = 0.109, *T*_max_ = 0.432	*l* = −22→22
3331 measured reflections	3 standard reflections every 120 min
1669 independent reflections	intensity decay: 1.0%

### Refinement

**Table:**

Refinement on *F*^2^	Secondary atom site location: difference Fourier map
Least-squares matrix: full	Hydrogen site location: difference Fourier map
*R*[*F*^2^ > 2σ(*F*^2^)] = 0.049	Only H-atom coordinates refined
*wR*(*F*^2^) = 0.143	*w* = 1/[σ^2^(*F*_o_^2^) + (0.0831*P*)^2^ + 0.169*P*] where *P* = (*F*_o_^2^ + 2*F*_c_^2^)/3
*S* = 1.10	(Δ/σ)_max_ = 0.012
1669 reflections	Δρ_max_ = 0.39 e Å^−^^3^
125 parameters	Δρ_min_ = −0.49 e Å^−^^3^
3 restraints	Extinction correction: *SHELXL97* (Sheldrick, 2008), Fc^*^=kFc[1+0.001xFc^2^λ^3^/sin(2θ)]^-1/4^
Primary atom site location: structure-invariant direct methods	Extinction coefficient: 0.0126 (16)

### Special details

**Table:**

Geometry. All e.s.d.'s (except the e.s.d. in the dihedral angle between two l.s. planes) are estimated using the full covariance matrix. The cell e.s.d.'s are taken into account individually in the estimation of e.s.d.'s in distances, angles and torsion angles; correlations between e.s.d.'s in cell parameters are only used when they are defined by crystal symmetry. An approximate (isotropic) treatment of cell e.s.d.'s is used for estimating e.s.d.'s involving l.s. planes.
Refinement. Refinement of *F*^2^ against ALL reflections. The weighted *R*-factor *wR* and goodness of fit *S* are based on *F*^2^, conventional *R*-factors *R* are based on *F*, with *F* set to zero for negative *F*^2^. The threshold expression of *F*^2^ > σ(*F*^2^) is used only for calculating *R*-factors(gt) *etc*. and is not relevant to the choice of reflections for refinement. *R*-factors based on *F*^2^ are statistically about twice as large as those based on *F*, and *R*- factors based on ALL data will be even larger.

### Fractional atomic coordinates and isotropic or equivalent isotropic displacement parameters (Å^2^)

**Table:**

	*x*	*y*	*z*	*U*_iso_*/*U*_eq_	
Cl1	0.64647 (11)	−0.35617 (13)	0.11963 (6)	0.0720 (4)	
Cl2	0.84843 (12)	0.33419 (16)	−0.08256 (4)	0.0716 (4)	
N1	0.8342 (3)	0.0359 (4)	0.16645 (11)	0.0476 (6)	
H11	0.872 (4)	−0.079 (6)	0.1920 (19)	0.057*	
H12	0.736 (5)	0.083 (5)	0.1868 (18)	0.057*	
H13	0.917 (5)	0.128 (6)	0.179 (2)	0.057*	
C1	0.7922 (3)	0.0096 (5)	0.09016 (13)	0.0441 (6)	
C2	0.7056 (4)	−0.1657 (5)	0.06338 (18)	0.0529 (7)	
C3	0.6664 (4)	−0.1873 (6)	−0.0093 (2)	0.0635 (9)	
H3	0.604 (5)	−0.303 (7)	−0.024 (2)	0.076*	
C4	0.7107 (4)	−0.0333 (6)	−0.05366 (16)	0.0626 (9)	
H4	0.681 (5)	−0.046 (6)	−0.100 (2)	0.075*	
C5	0.7943 (4)	0.1398 (5)	−0.02646 (15)	0.0534 (7)	
C6	0.8371 (4)	0.1651 (5)	0.04598 (15)	0.0474 (6)	
H6	0.905 (4)	0.288 (5)	0.065 (2)	0.057*	
O1	0.0902 (3)	0.3197 (4)	0.19565 (15)	0.0697 (7)	
H1A	0.200 (4)	0.301 (6)	0.206 (3)	0.084*	
H1B	0.067 (5)	0.413 (6)	0.223 (2)	0.084*	
Cl3	0.50259 (9)	0.21839 (12)	0.22100 (4)	0.0565 (3)	

### Atomic displacement parameters (Å^2^)

**Table:**

	*U*^11^	*U*^22^	*U*^33^	*U*^12^	*U*^13^	*U*^23^
Cl1	0.0655 (6)	0.0603 (5)	0.0883 (6)	−0.0101 (3)	0.0009 (5)	0.0022 (4)
Cl2	0.0671 (6)	0.0981 (7)	0.0499 (4)	0.0101 (4)	0.0085 (4)	0.0145 (4)
N1	0.0485 (13)	0.0551 (13)	0.0379 (10)	0.0017 (11)	−0.0002 (9)	−0.0007 (10)
C1	0.0377 (13)	0.0528 (14)	0.0402 (11)	0.0065 (11)	−0.0015 (10)	−0.0062 (11)
C2	0.0400 (13)	0.0555 (16)	0.0615 (17)	0.0031 (12)	−0.0004 (13)	−0.0084 (13)
C3	0.0500 (17)	0.0694 (19)	0.0677 (19)	0.0019 (15)	−0.0065 (15)	−0.0270 (17)
C4	0.0522 (16)	0.086 (2)	0.0470 (14)	0.0111 (16)	−0.0034 (13)	−0.0205 (15)
C5	0.0436 (14)	0.076 (2)	0.0405 (13)	0.0130 (13)	0.0032 (12)	−0.0027 (13)
C6	0.0415 (13)	0.0578 (15)	0.0419 (13)	0.0044 (12)	0.0009 (11)	−0.0054 (12)
O1	0.0571 (14)	0.0722 (16)	0.0776 (15)	−0.0017 (11)	−0.0004 (12)	−0.0175 (12)
Cl3	0.0529 (5)	0.0671 (5)	0.0484 (4)	−0.0018 (3)	0.0015 (3)	−0.0101 (3)

### Geometric parameters (Å, °)

**Table:**

Cl1—C2	1.731 (3)	C3—C4	1.377 (6)
Cl2—C5	1.735 (3)	C3—H3	0.92 (4)
N1—C1	1.461 (3)	C4—C5	1.362 (5)
N1—H11	0.92 (4)	C4—H4	0.88 (4)
N1—H12	0.94 (4)	C5—C6	1.390 (4)
N1—H13	0.88 (4)	C6—H6	1.00 (4)
C1—C2	1.382 (4)	O1—H1A	0.85 (3)
C1—C6	1.383 (4)	O1—H1B	0.83 (3)
C2—C3	1.388 (5)		
			
C1—N1—H11	117 (2)	C4—C3—H3	124 (3)
C1—N1—H12	111 (2)	C2—C3—H3	116 (3)
H11—N1—H12	105 (3)	C5—C4—C3	120.1 (3)
C1—N1—H13	114 (2)	C5—C4—H4	121 (3)
H11—N1—H13	104 (3)	C3—C4—H4	119 (3)
H12—N1—H13	105 (3)	C4—C5—C6	121.3 (3)
C2—C1—C6	121.2 (3)	C4—C5—Cl2	120.0 (2)
C2—C1—N1	120.1 (3)	C6—C5—Cl2	118.8 (3)
C6—C1—N1	118.7 (3)	C1—C6—C5	118.2 (3)
C1—C2—C3	119.2 (3)	C1—C6—H6	121 (2)
C1—C2—Cl1	120.5 (2)	C5—C6—H6	121 (2)
C3—C2—Cl1	120.3 (3)	H1A—O1—H1B	104 (4)
C4—C3—C2	120.0 (3)		
			
C6—C1—C2—C3	1.3 (4)	C3—C4—C5—C6	0.2 (5)
N1—C1—C2—C3	179.8 (3)	C3—C4—C5—Cl2	−179.5 (3)
C6—C1—C2—Cl1	−178.6 (2)	C2—C1—C6—C5	−0.6 (4)
N1—C1—C2—Cl1	−0.2 (4)	N1—C1—C6—C5	−179.1 (3)
C1—C2—C3—C4	−1.3 (5)	C4—C5—C6—C1	−0.2 (4)
Cl1—C2—C3—C4	178.7 (3)	Cl2—C5—C6—C1	179.6 (2)
C2—C3—C4—C5	0.5 (5)		

### Hydrogen-bond geometry (Å, °)

**Table:**

*D*—H···*A*	*D*—H	H···*A*	*D*···*A*	*D*—H···*A*
N1—H11···Cl3^i^	0.92 (4)	2.24 (4)	3.123 (3)	162 (3)
N1—H12···Cl3	0.94 (4)	2.16 (4)	3.099 (3)	172 (3)
N1—H13···O1^ii^	0.88 (4)	1.82 (4)	2.699 (4)	175 (4)
O1—H1A···Cl3	0.85 (3)	2.37 (3)	3.212 (3)	172 (4)
O1—H1B···Cl3^iii^	0.83 (3)	2.34 (3)	3.158 (3)	169 (4)

Symmetry codes: (i) −*x*+3/2, *y*−1/2, −*z*+1/2; (ii) *x*+1, *y*, *z*; (iii) −*x*+1/2, *y*+1/2, −*z*+1/2.
